# Characterization of fetal microchimeric immune cells in mouse maternal hearts during physiologic and pathologic pregnancies

**DOI:** 10.3389/fcell.2023.1256945

**Published:** 2023-09-22

**Authors:** Ryan C. V. Lintao, Ananth Kumar Kammala, Enkhtuya Radnaa, Mohamed Bettayeb, Kathleen L. Vincent, Igor Patrikeev, Jerome Yaklic, Elizabeth A. Bonney, Ramkumar Menon

**Affiliations:** ^1^ Division of Basic Science and Translational Research, Department of Obstetrics and Gynecology, The University of Texas Medical Branch at Galveston, Galveston, TX, United States; ^2^ Department of Biochemistry and Molecular Biology, College of Medicine, University of the Philippines Manila, Manila, Philippines; ^3^ Biomedical Engineering and Imaging Sciences Group, The University of Texas Medical Branch at Galveston, Galveston, TX, United States; ^4^ Department of Obstetrics and Gynecology, The University of Texas Medical Branch at Galveston, Galveston, TX, United States; ^5^ Department of Obstetrics, Gynecology, and Reproductive Sciences, Larner College of Medicine, The University of Vermont, Burlington, VT, United States

**Keywords:** microchimerism, fetal microchimerism, preterm birth, cardiovascular risk, infection, fetal cells, pregnancy, ascending infection

## Abstract

**Introduction:** During pregnancy, fetal cells can be incorporated into maternal tissues (fetal microchimerism), where they can persist postpartum. Whether these fetal cells are beneficial or detrimental to maternal health is unknown. This study aimed to characterize fetal microchimeric immune cells in the maternal heart during pregnancy and postpartum, and to identify differences in these fetal microchimeric subpopulations between normal and pregnancies complicated by spontaneous preterm induced by ascending infection.

**Methods:** A Cre reporter mouse model, which when mated with wild-type C57BL/6J females resulted in cells and tissues of progeny expressing red fluorescent protein tandem dimer Tomato (mT+), was used to detect fetal microchimeric cells. On embryonic day (E)15, 10^4^ colony-forming units (CFU) *E. coli* was administered intravaginally to mimic ascending infection, with delivery on or before E18.5 considered as preterm delivery. A subset of pregnant mice was sacrificed at E16 and postpartum day 28 to harvest maternal hearts. Heart tissues were processed for immunofluorescence microscopy and high-dimensional mass cytometry by time-of-flight (CyTOF) using an antibody panel of immune cell markers. Changes in cardiac physiologic parameters were measured up to 60 days postpartum via two-dimensional echocardiography.

**Results:** Intravaginal *E. coli* administration resulted in preterm delivery of live pups in 70% of the cases. mT + expressing cells were detected in maternal uterus and heart, implying that fetal cells can migrate to different maternal compartments. During ascending infection, more fetal antigen-presenting cells (APCs) and less fetal hematopoietic stem cells (HSCs) and fetal double-positive (DP) thymocytes were observed in maternal hearts at E16 compared to normal pregnancy. These HSCs were cleared while DP thymocytes persisted 28 days postpartum following an ascending infection. No significant changes in cardiac physiologic parameters were observed postpartum except a trend in lowering the ejection fraction rate in preterm delivered mothers.

**Conclusion:** Both normal pregnancy and ascending infection revealed distinct compositions of fetal microchimeric immune cells within the maternal heart, which could potentially influence the maternal cardiac microenvironment via (1) modulation of cardiac reverse modeling processes by fetal stem cells, and (2) differential responses to recognition of fetal APCs by maternal T cells.

## Introduction

Fetal microchimerism refers to the presence of a small number of fetal cells in the maternal body (O'Donoghue, 2008). During pregnancy, fetal cells such as fetal stem cells or immune cells can cross the placenta and enter the maternal circulation. These fetal cells can then migrate and integrate into various maternal tissues and organs (Bianchi et al., 2021). As a result, the mother may retain a small population of cells that originated from her fetus. These cells can persist in the maternal body for years or even decades after pregnancy (Bianchi et al., 1996; Chan and Nelson, 2013), but may also be impacted by the maternal immune system (Bonney and Matzinger, 1997; Bonney and Onyekwuluje, 2003). The relationship between maternal health outcomes and fetal microchimerism is an area of active research and ongoing investigation.

While the precise mechanisms and implications are not yet fully understood, some studies have suggested potential associations between fetal microchimerism and certain maternal health conditions. Fetal cells that persist in maternal tissues have been proposed to possess regenerative properties and have the potential to differentiate into various cell types, thus contributing to tissue repair and regeneration (Mahmood and O'Donoghue, 2014; Kara et al., 2012). They were also implicated in the development or modulation of autoimmune diseases such as systemic sclerosis (Saw et al., 2004), rheumatoid arthritis (Kekow et al., 2013), and systemic lupus erythematosus (Johnson et al., 2001; Kekow et al., 2013). The characteristics of fetal microchimeric cells in women with normal pregnancy and pregnancies complicated by preterm birth (delivery of an offspring before 37 weeks of gestation), specifically spontaneous preterm births of unknown etiologies, are expected to differ.

Spontaneous preterm birth, predominantly associated with ascending infection, results in significant perinatal morbidity and mortality (Blencowe et al., 2012; Cao et al., 2022) and can lead to long-term neurodevelopmental impairment (Hee Chung et al., 2020) and chronic health problems to the baby (Luu et al., 2016). Preterm delivery also presents substantial risk to the mother for immediate complications such as hemorrhage, infection and admission into intensive care unit (Reddy et al., 2015), postnatal depression (Leahy-Warren et al., 2020), and chronic conditions such as hypertension, type 2 diabetes mellitus, and hypercholesterolemia (Tanz et al., 2019). Women who delivered preterm are also at higher risk for ischemic heart disease with an adjusted hazard ratio of 2.47 (95% CI, 2.16–2.82) 10 years following delivery, with further increases in risk with every additional preterm delivery (Crump et al., 2020). Although an association was observed between high-risk blood pressure pattern in women who delivered preterm and coronary artery calcification, mechanisms linking preterm delivery and future maternal cardiovascular diseases are not fully understood (Catov et al., 2018).

Due to their ability to persist long-term and their differential responses (i.e., repair vs. autoimmune) depending on the context of pregnancy, we hypothesize that fetal microchimeric cells can migrate to the maternal heart, and that their phenotype and functional potential may vary with normal and abnormal pregnancy, including preterm birth. This study focused on characterizing fetal microchimeric immune cells in a cyclic recombinase (Cre)-reporter mouse model of ascending infection and preterm birth. By utilizing high-dimensional mass cytometry by time-of-flight (CyTOF), different subpopulations of fetal microchimeric cells were identified in the maternal heart during pregnancy and after delivery. The studies herein serve as a crucial step in understanding function and potential impact of these cells on maternal cardiovascular health.

## Materials and methods

### Animal care

**FIGURE 1:**
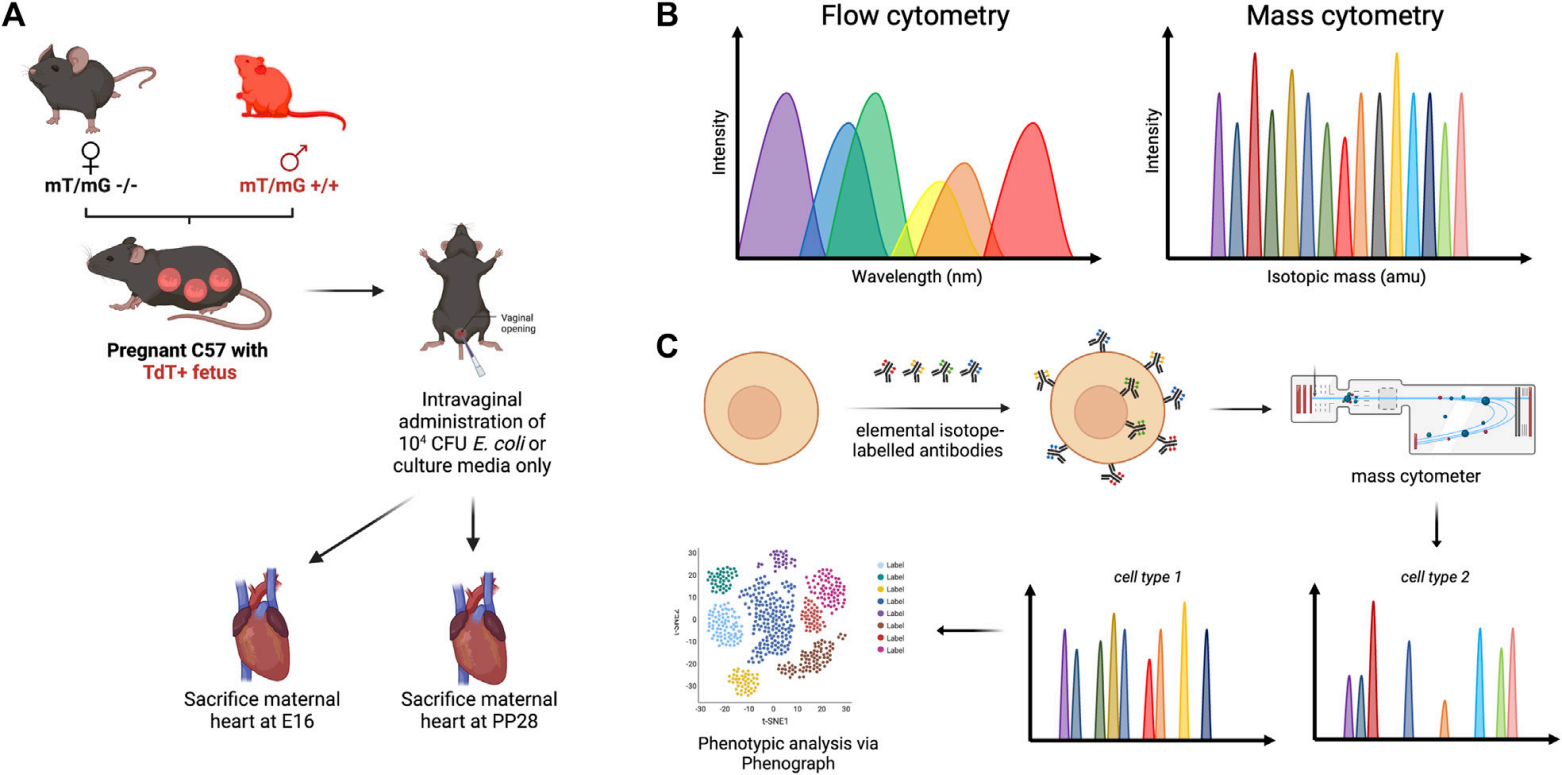
Methodology framework for high-dimensional mass cytometry. **(A)** Male homozygous mT/mG +/+ C57BL/6J mice were mated with wild-type females, which resulted in progeny cells that express mT+. Pregnant mice were treated intravaginally with 10^4^ CFU *E. coli* to mimic ascending infection, with sterile nutrient broth serving as negative control. **(B)** Both flow cytometry and mass cytometry utilize cell labeling with antibodies to facilitate single-cell analysis, although fewer spectral overlap is observed with mass cytometry. **(C)** Molecules of interest were tagged with elemental isotope-labelled antibodies. Following nebulization and injection into a mass analyzer, the ionized metals were accelerated through a vacuum chamber and were analyzed based on time of flight.

A cyclic recombinase (Cre)-reporter mouse model was used to study fetal microchimeric cells as detailed by Sheller-Miller et al. (2019), wherein all cells and tissues of progeny expressed mT+ (Sheller-Miller et al., 2019). Eight-to 12-week old homozygous transgenic B6.129(Cg)-*Gt(ROSA)26Sor* ^*tm4(ACTB-mT+,-EGFP)Luo/J*^ (mT/mG) males as previously described by Muzumdar et al. (Muzumdar et al., 2007) (Strain #007676, The Jackson Laboratory, Bar Harbor, ME) were mated with wild type C57BL/6J females (Strain #000664, The Jackson Laboratory, Bar Harbor, ME) of the same age (Figure 1A). The following morning, females observed with vaginal plug (indicating gestational day 0.5 (E0.5) were housed separately from the males. A cohort of nonpregnant mice were maintained for ultrasonographic studies. The mice were housed in a temperature- and humidity-controlled animal facility with 12-h light and dark cycles, and regular chow and drinking solutions provided *ad libitum*. All animal procedures were approved by the Institutional Animal Care and Use Committee (IACUC) at the University of Texas Medical Branch at Galveston under protocol number 0411077E.

### *Escherichia coli* (*E. coli)* culture

A day prior to experiment, a bacterial culture of ATCC 12014 *Escherichia coli* O55:K59(B5):H– (Lot #496291, ThermoFisher Scientific Remel Products, San Diego, CA, United States) was inoculated in 200 mL sterile nutrient broth (Difco, Cat. # 234000, BD Biosciences, Franklin Lakes, NJ, United States) and cultured for 16 h at 37°C with shaking. Using a spectrophotometer (D30 BioPhotometer, Eppendorf, Hamburg, Germany), OD_600_ value was measured and colony forming units per milliliter (CFU/mL) was calculated based on the standard curve generated by Spencer et al. (2021).

### Model of ascending infection-induced preterm birth

At E15, pregnant mice were anesthetized via isoflurane inhalation. Based on CFU/mL calculation, 10^4^ CFU of *E. coli* in 40 µL nutrient broth was administered vaginally to pregnant mice (*n* = 13) using a blunted 200 µL pipette tip (Figure 1A). The same volume of sterile nutrient broth was used in age-matched nonpregnant (*n* = 3) and pregnant control groups (*n* = 11). The timing of delivery was monitored using live cameras (Shenzen Wansview Technology, Shenzen, China). Delivery of the first pup on or before E18.5 was considered preterm. A subset of pregnant C57BL/6J mice (3 nutrient broth-treated, 5 *E. coli*-treated) were euthanized at E16 (1 day post-treatment) *via* carbon dioxide inhalation according to IACUC and the American Veterinary Medical Association guidelines, while the remaining mice (5 nutrient broth-treated, 5 *E. coli*-treated) were euthanized at postpartum day 28 (PP28).

### Immunofluorescent imaging for mT + signal in maternal heart tissues

Placental, and maternal uterine, heart, lung, kidney and brain specimens were collected after euthanization. Visible placental fragments were gently removed, and the uterine lining was gently scraped to remove any adherent placental cells. The samples were then fixed in 4% paraformaldehyde overnight at 4°C. After fixation, the samples were washed twice with 1× phosphate-buffered saline (PBS) and subsequently placed in a 15% sucrose solution overnight at 4°C. They were then transferred to a 30% sucrose solution and stored at 4°C until embedding in optimal cutting temperature (OCT) compound. Multiple 5 mm-sections were then incubated at room temperature for 30 min, followed by two washes in water to remove the OCT. The sections were incubated with DAPI for 2 min at room temperature, followed by two washes in water. To minimize autofluorescence, the tissues were treated with TrueVIEW Autofluorescence Quenching Kit (Vector Laboratories, Burlingame, CA) for 10 s, and washed twice with 1X Tris-buffered saline + Tween 20 (TBS-T). Subsequently, the slides were air dried at room temperature for 10 min and mounted using Mowiol 4–88 mounting medium.

### Single-cell preparation from maternal heart tissues

To prepare the heart tissues, excess fat was carefully removed using fine forceps, and the tissues were washed with cold 1× phosphate-buffered saline (pH 7.4) and placed in RPMI 1,640 with 10% fetal bovine serum and 1% penicillin-streptomycin. The heart tissues were then transferred to Accutase cell detachment solution (Corning, Corning, NY, United States) and cut into small pieces using fine scissors. After digestion for 60 min with gentle rocking at 37°C, the tissue samples were strained through a 70-µm cell strainer. The homogenates were washed twice with RPMI 1640 with 10% fetal bovine serum and 1% penicillin-streptomycin and centrifuged at 300 g for 5 min at 20°C. To remove erythrocytes, the cell pellets were resuspended in 1.0 mL red blood cell lysis buffer (BioLegend, San Diego, CA, United States), incubated for 10 min at room temperature, and diluted to 10 mL with RPMI 1640 with 10% fetal bovine serum and 1% penicillin-streptomycin. The samples were centrifuged at 300 g for 5 min at 20°C, and the resulting cell pellets were then resuspended in 0.5 mL commercial cell freezing media until use.

### High-dimensional single-cell profiling of maternal heart tissues via mass cytometry by time-of-flight (CyTOF)

#### Antibodies

Basics of mass cytometry were discussed in Figures 1B, C. The antibodies used in this study were obtained either from the Flow Cytometry and Cellular Imaging Core Facility at The University of Texas MD Anderson Cancer Center in Houston, TX, United States, or custom-conjugated using the Maxpar antibody conjugation kit (Standard BioTools, Markham, ON, Canada) following the manufacturer’s protocol. A comprehensive list of the antibodies utilized can be found in Supplementary Table S1. Following conjugation with their respective metal labels, the percentage yield was determined by measuring the absorbance at 280 nm using a Nanodrop 2000 spectrophotometer (Thermo Scientific, Wilmington, DE, United States). The antibodies were then diluted to a concentration of 0.3 mg/mL using Candor phosphate-buffered saline (PBS) antibody stabilization solution (Candor Bioscience GmbH, Wangen, Germany) and stored at 4°C for future use.

#### Antibody staining

Single cells suspension samples were resuspended in Maxpar staining buffer for 10 min at room temperature on a shaker to block Fc receptors. Cells were mixed with a cocktail of metal-conjugated surface marker antibodies, yielding 500 μL final reaction volumes, and stained at room temperature for 30 min on a shaker. Following staining, cells were washed twice with Maxpar staining buffer. Next, cells were permeabilized with 4°C Max Perm Buffer for 10 min at 4 °C. Cells were then washed twice in Maxpar staining buffer to remove the remaining Max Perm. They were stained with intracellular antibodies in 500 μL for 30 min at room temperature on a shaker. Samples were then washed twice in Maxpar staining buffer. Cells were incubated overnight at 4°C with 1 mL of 1:4,000 191/193Ir DNA intercalator (Standard BioTools, Markham, ON, Canada) diluted in Maxpar Fix/Perm overnight. The following day, cells were washed once with Maxpar staining buffer and then two times with deionized water (ddH_2_O).

#### Mass cytometry

Prior to analysis, the cell pellet stained with antibodies and intercalated with DNA intercalator was resuspended in ddH_2_O containing polystyrene normalization beads. The normalization beads contained lanthanum-139, praseodymium-141, terbium-159, thulium-169, and lutetium-175, following the method described by Finck et al. (2013). Stained cells were then analyzed using a CyTOF 2 instrument (Standard BioTools Inc, Markham, ON, Canada) equipped with a Super Sampler sample introduction system (Victorian Airship & Scientific Apparatus, Alamo, CA, United States). The event rate was set to 200 to 300 cells per second. To ensure accurate data normalization, all mass cytometry files were normalized using the mass cytometry data normalization algorithm *premessa* available at https://github.com/ParkerICI/premessa/.

#### Data analysis

The Flow cytometry standard (.fcs) files were analyzed using FlowJo v10.9.0 (FlowJo LLC, Ashland, OR, United States). To ensure data quality, intact live single cells were manually gated using the Standard BioTools (Markham, ON, Canada) clean-up procedure. Each sample was assigned a unique Sample ID, and all samples were combined into a single.fcs file for further analysis. The concatenated file was subjected to *t*-distributed stochastic neighbor embedding (t-SNE) in FlowJo, utilizing equal numbers of cells from both the normal pregnancy and ascending infection mouse groups, along with all phenotypic markers. The following settings were used: iterations (3,000), perplexity (Motomura et al., 2015), learning rate (eta) (8,960), KNN algorithm (exact, vantage point tree), and gradient algorithm (Barnes-Hut). To explore the phenotypic diversity of immune cell populations in the maternal hearts of different mouse groups, we employed the Phenograph K-nearest-neighbor density-based clustering algorithm. This unsupervised clustering analysis was performed on the data from single cells (Levine et al., 2015). To visualize the continuum of phenotypic cell populations, the output was organized using the Cluster Explorer plug-in, which generated an interactive cluster profile graph and heatmap, and displayed the cluster populations on a t-SNE plot.

### Two-dimensional murine echocardiography

Echocardiography was performed using a Vevo 2,100 high-resolution ultrasound system (VisualSonics, Toronto, ON, Canada) to measure changes in cardiac and physiology over time in a non-invasive manner, with the protocol adapted from Herrera et al. (2018) (Herrera et al., 2019). A total of 9 mice (*n* = 3 for normal pregnancy, *n* = 3 for ascending infection, *n* = 3 for nonpregnant control) were used for the study, evaluated on days 7, 14, 21, 28, and 60 postpartum. On the day of evaluation, mice were anesthetized with 1%–2% isoflurane in oxygen delivered through a nosecone using a controlled-delivery anesthetic machine and were placed supine on a warmed platform with integrated electrocardiographic monitoring of heart rate, core temperature, and respiration. A sterile ophthalmic ointment (Artificial Tears, Akorn, Lake Forest, IL) was applied to the eyes to prevent them from drying out. Animal temperature was monitored with a rectal temperature probe, while pulse rate and respiration rate were monitored via electrode gel-lubricated electrode pads on the platform. Chest and abdominal hair were removed from the mice with a chemical hair removal lotion (Nair, Church & Dwight, Ewing, NJ). A 30-MHz transducer lubricated with high-viscosity ultrasound gel was used to evaluate the following parameters of cardiac function: heart rate (beats/min), cardiac output (mL/min) and ejection fraction (%). From the parasternal long axis (PLAX) view, the left ventricular outflow diameter and the blood flow through the aorta per unit time were measured. Cardiac output was calculated as the product of cross-sectional area × velocity time integral × heart rate. Systolic function was assessed from the parasternal short axis (PSAX) view by measuring end diastolic diameter (EDD) and end systolic diameter (ESD) using M-mode. Left ventricular ejection fraction was calculated as (EDD^3^-ESD^3^)/EDD^3^. Scanning took approximately 30 min per animal. Data was analyzed via one-way analysis of variance (ANOVA) with *post hoc* Tukey’s honestly significant difference (HSD) test using GraphPad Prism 9.5.0.

## Results

### Fetal microchimeric cells migrate up to the maternal heart

**FIGURE 2:**
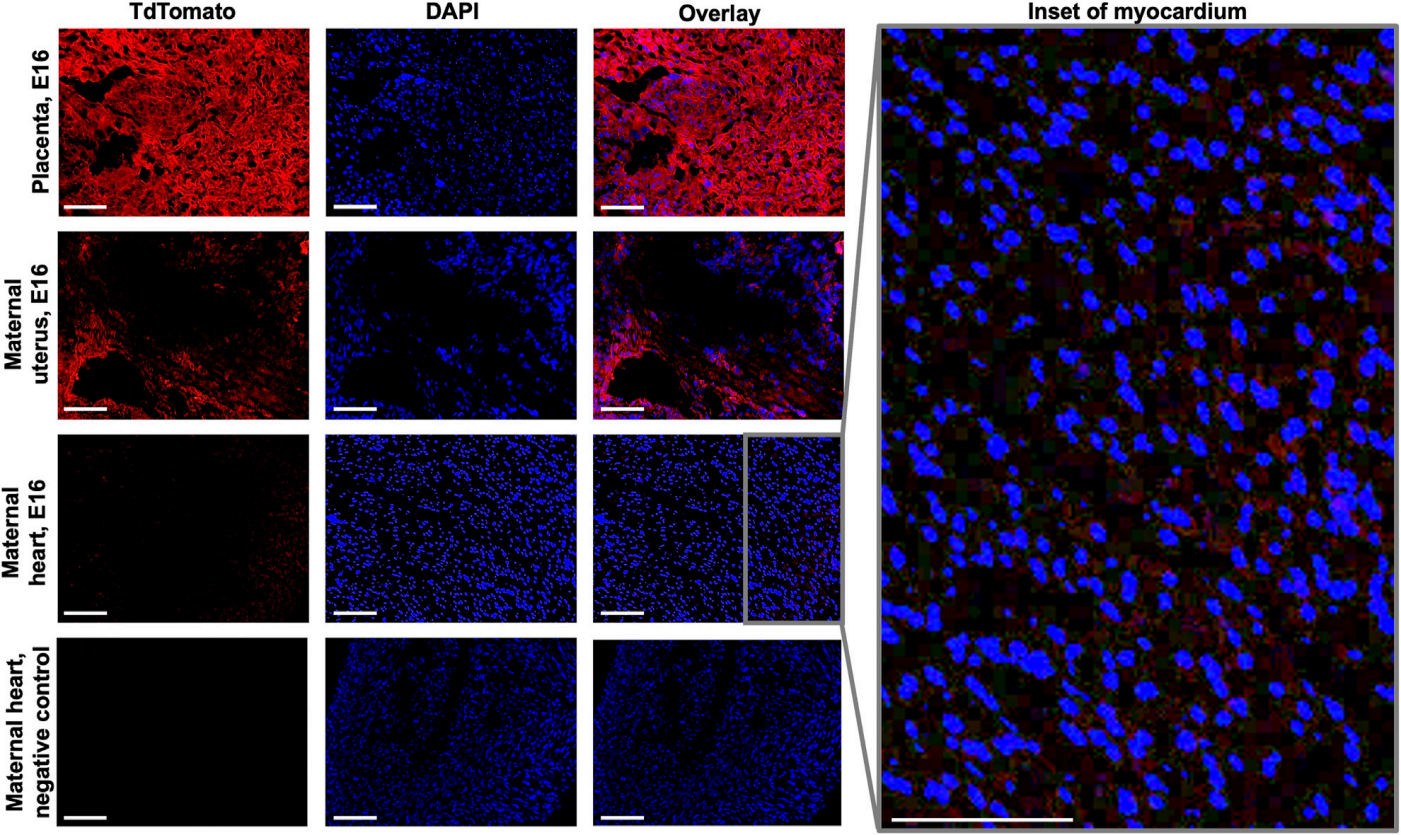
Fluorescence imaging of fetal and maternal tissues at E16. Sections of placenta, maternal uterine and maternal heart tissues at E16 were viewed under both Texas Red and DAPI filters to visualize mT+ and nuclear stain, respectively. Inset shows increased magnification of overlaid maternal heart tissue image. Negative control used was maternal heart from wild-type female C57BL/6J mated with wild-type male C57BL/6J. Scale bar: 50 µm.

Immunofluorescence microscopy was employed to examine the presence of fetal microchimeric cells at the maternal-fetal interface and assess their distribution in the maternal heart. Tissues collected on E16 were subjected to this analysis, with the aim of identifying mT + cells (Figure 2). As anticipated, the placenta derived from the fetus displayed widespread expression of mT+. In the maternal uterus, mT + cells were also observed, albeit mT + -expressing cells were detected in the maternal hearts on E16, although their fluorescence intensity was minimal (as depicted in the inset of Figure 2). mT + cells were also found in other maternal organs such as lungs and kidneys, but interestingly not in maternal brain (Supplementary Figure S1). To rule out the possibility of autofluorescence, a negative control (maternal heart at E16 from wild-type female C57BL/6J mated with wild-type male C57BL/6J) which exhibited no red fluorescence.

### Cell subpopulations in maternal hearts during normal pregnancy and during ascending infection

**FIGURE 3:**
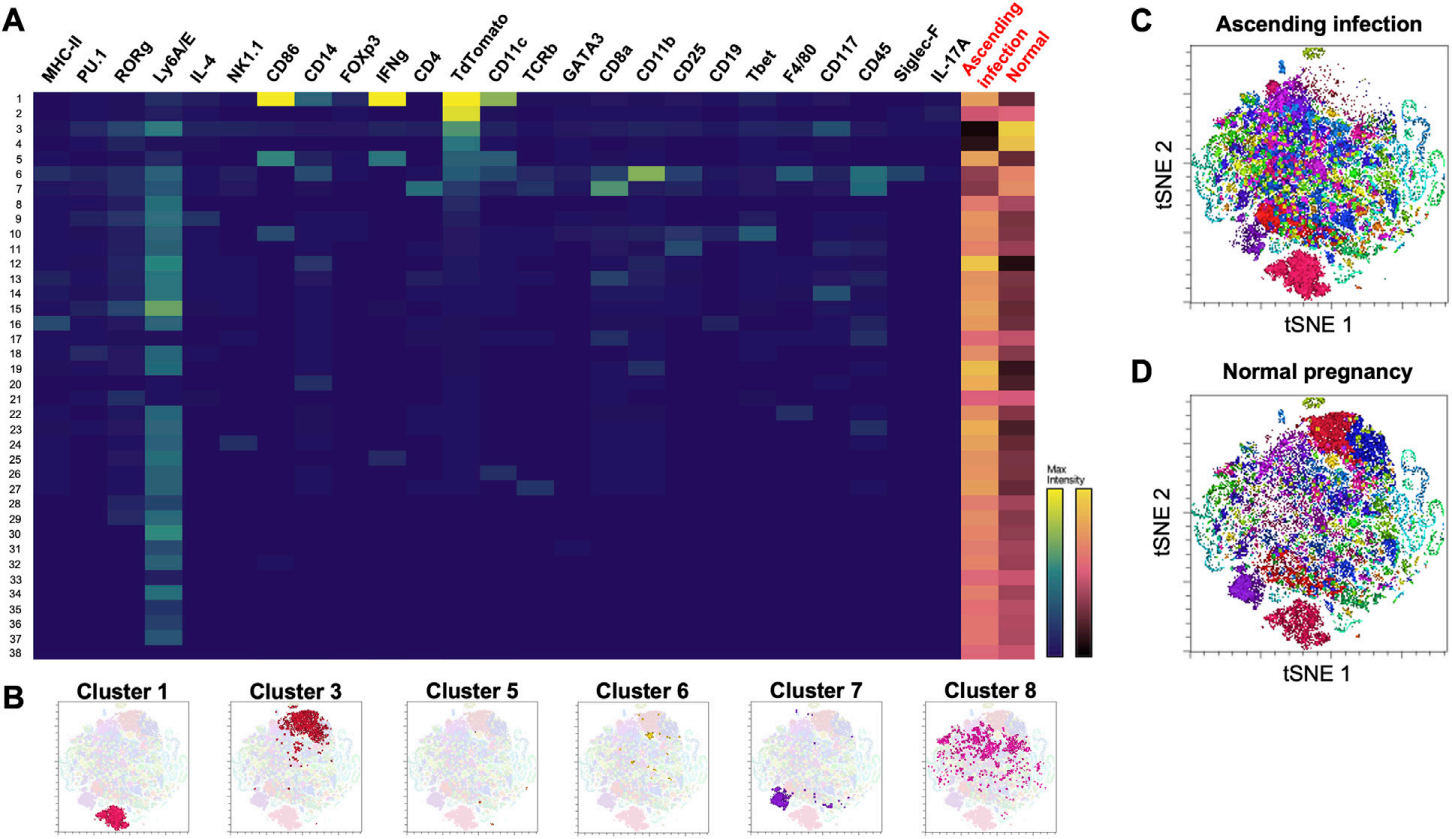
Phenograph cluster profiles of cells found in maternal heart at E16 during normal pregnancy and ascending infection. **(A)** Heatmap of 38 clusters generated by Phenograph, the various cell markers present, and their frequency in both ascending infection and normal pregnancy. Columns with black labels show the expression levels of a certain cell marker in each cluster, with yellow as the maximum marker intensity and blue as the minimum marker intensity. Columns with red labels at the rightmost side portray the frequency of each cluster in ascending infection and normal pregnancy, with gold as the highest frequency and black as the lowest frequency. **(B)** Location of clusters 1, 3, 5, 6, 7, and 8 in the t-SNE plot. **(C)** Cell clusters present in ascending infection superimposed on t-SNE plot. **(D)** Cell clusters present in normal pregnancy superimposed on t-SNE plot.

Among the 38 clusters (*k* = 183) generated using Phenograph (Figure 3A), 8 clusters were found to express mT+, representing approximately 28.76% of the cell population isolated from maternal heart. This frequency was most likely an overestimation of the fetal microchimeric population [usual population is < 1 in 10,000 cells (Fjeldstad et al., 2020)] given that the single cell preparation was prepared with 70 µm filter, which would deplete most 100 µm-long rod-shaped maternally-derived cardiomyocytes (Miranda et al., 2023). Cluster 3 exhibited the highest frequency among the mT + -positive cells at 7.85%, followed by cluster 1 at 6.77%, and cluster 8 at 5.78%. A summary of the identifiable mT + -expressing clusters based on high-dimensional mass cytometry data is presented in Table 1 and visualized in Figure 3B. Cluster 1 expressed high CD14, CD11c, CD86 and IFN-γ which is indicative of a subpopulation of CD11c^+^ M1 macrophages. Cluster 3 expressed CD117 (c-kit) and Ly6A/E (Sca-1) and showed negative expression for lineage markers CD4, CD8, and CD11b (Mac-1), consistent with the Lineage^−^Sca-1^+^c-kit^+^ (LSK^+^) phenotype of murine hematopoietic cells. Cluster 5 was similar to Cluster 1, but with lower expression of CD14, pointing to an activated conventional dendritic cell phenotype. Cluster 6 expressed the monocyte-macrophage marker CD14, the myeloid marker CD11b, and F4/80, while being negative for macrophage polarization markers, indicating a non-activated macrophage phenotype. Cluster 7 expressed both CD4 and CD8, and low T cell receptor, characteristic of double-positive thymocytes. Cluster 8 was similar to cluster 3 except the former lacked expression of CD117 (c-kit), pointing to a Lineage^−^Sca-1^+^c-kit^-^ (LSK^−^) phenotype of murine hematopoietic cells called very small embryonic-like (VSEL) cells. Interestingly, clusters 2 and 4 did not exhibit expression of CD45 or other immune markers, suggesting a non-hematopoietic origin for these clusters. Among the identifiable clusters in Table 1, clusters 1, 5 and 8 showed an increase in frequency in ascending infection, whereas clusters 3, 6, and 7 displayed a decrease in frequency (Figures 3C, D).

**TABLE 1:** mT + cell clusters in the maternal heart at E16.

Cluster	Phenotype	Markers	Changes in pathologic vs. normal pregnancy	Reference
1	CD11c^+^ M1 macrophages	CD14^high^, CD11c^+^, CD86^+^, IFN-γ^high^	↑↑	Munder et al. (1998), Motomura et al. (2015), Zhou et al. (2017)
3	LSK^+^ hematopoietic stem cells	CD117^+^, Ly6A/E^+^, CD4^−^, CD8^−^, CD11b^-^	↓↓	Challen et al. (2009)
5	Activated conventional dendritic cells	CD14^low^, CD11c^+^, CD86^+^, IFN-γ^high^	↑↑	Vremec et al. (2007), Kim and Kim (2019)
6	M0 (non-activated) macrophages	CD45^+^, CD14^+^, CD11b^+^, F4/80^+^	↓	Dos Anj and os Cassado (2017)
7	Double-positive thymocytes	CD45^+^, TCRb^low^, CD4^+^, CD8^+^	↓	Brodeur et al. (2009), Li et al. (2021)
8	Very small embryonic-like (VSEL) cells	CD117^-^, Ly6A/E^+^, CD4^−^, CD8^−^, CD11b^-^	↑	Kumar et al. (2008), Szade et al. (2013)

### Fetal microchimeric cells persist in postpartum maternal hearts

**FIGURE 4:**
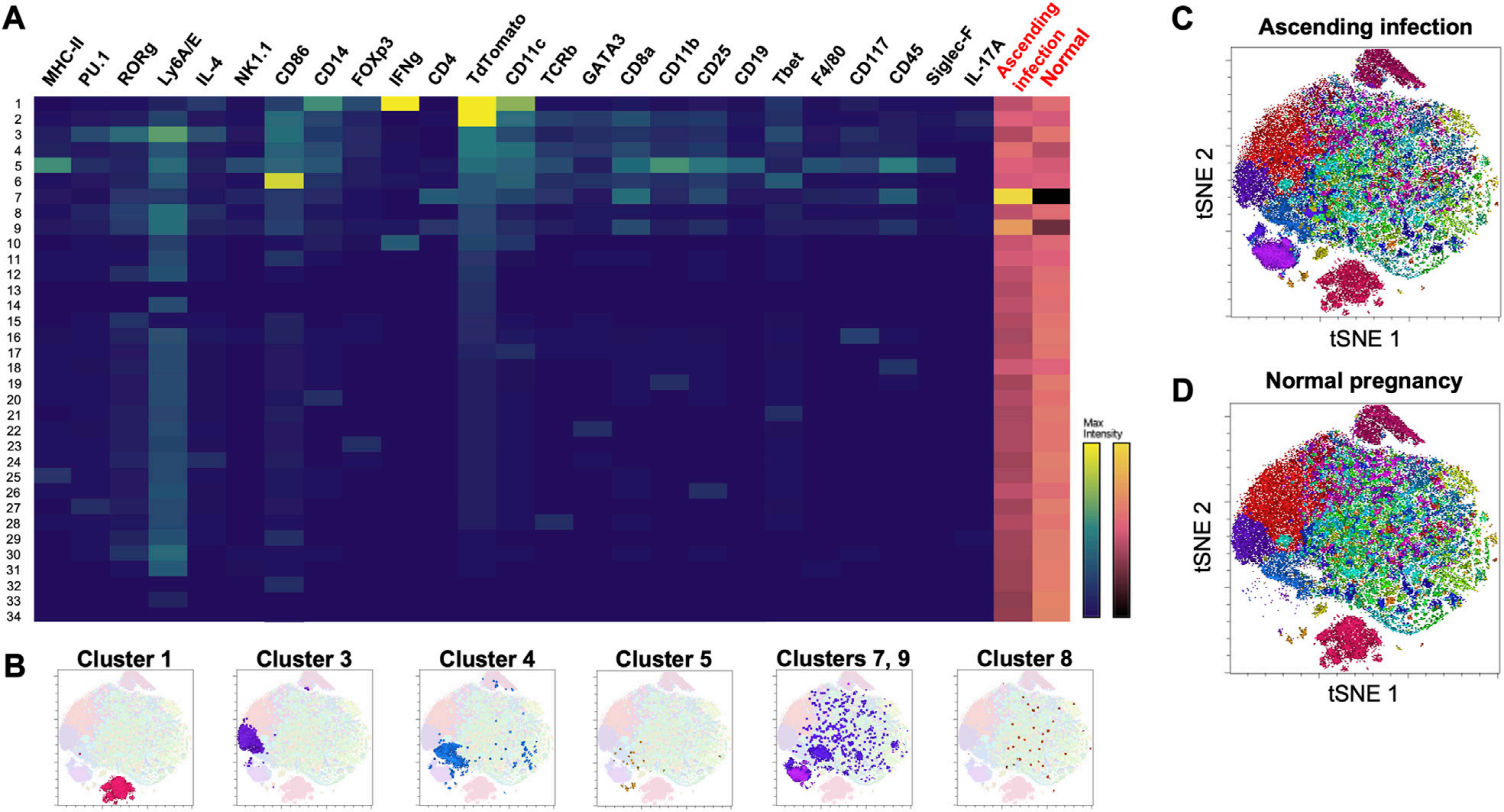
Phenograph cluster profiles of cells found in maternal heart at postpartum day 28 following normal pregnancy and ascending infection. **(A)** Heatmap of 34 clusters generated by Phenograph, the various cell markers present, and their frequency in both ascending infection and normal pregnancy. Columns with black labels show the expression levels of a certain cell marker in each cluster, with yellow as the maximum marker intensity and blue as the minimum marker intensity. Columns with red labels at the rightmost side portray the frequency of each cluster in ascending infection and normal pregnancy, with gold as the highest frequency and black as the lowest frequency. **(B)** Location of clusters 1, 3, 4, 5, 7, 8, and 9 in the t-SNE plot. **(C)** Cell clusters present in ascending infection superimposed on t-SNE plot. **(D)** Cell clusters present in normal pregnancy superimposed on t-SNE plot.

Thirty-four clusters were generated using Phenograph, 10 of which expressed TdTomato (Figure 4A). Table 2 shows the identifiable mT + -expressing cell clusters, while Figure 4B shows the cluster profiles in both ascending infection and normal pregnancy. Similar to E16, CD11c^+^ M1 macrophages (Cluster 1), LSK^+^ hematopoietic stem cells (Cluster 3), activated conventional dendritic cells (Cluster 4), double-positive thymocytes (Clusters 7 and 9) and very small embryonic-like (VSEL) cells (Cluster 8) were identified based on expression of similar markers. Interestingly, Cluster 5 expressed CD19, MHC-II, CD11b and CD25 which are cell markers for CD25^+^ B cells. Clusters 1, 3 and 8 showed a decrease in frequency with ascending infection, while clusters 4, 5, 7, and 9 showed an increase, with the latter two exhibiting marked increase in frequency (Figures 4C, D).

**TABLE 2:** mT + -expressing cell clusters in the maternal heart at PP28.

Cluster	Phenotype	Markers	Change in pathologic vs. normal pregnancy	Reference
1	CD11c^+^ M1 macrophages	CD14^high^, CD11c^+^, CD86^+^, IFN-γ^high^	↓	Munder et al. (1998), Motomura et al. (2015), Zhou et al. (2017)
3	LSK^+^ hematopoietic stem cells	CD117^+^, Ly6A/E^+^, CD4^−^, CD8^−^, CD11b^-^	↓	Challen et al. (2009)
4	Activated conventional dendritic cells	CD14^low^, CD11c^+^, CD86^+^, IFN-γ^high^	↑	Vremec et al. (2007), Kim and Kim (2019)
5	CD25^+^ B cells	CD45^+^, CD19^+^, MHCII^+^, CD25^+^, CD11b^+^	↑	Amu et al. (2010), Ma et al. (2012)
7	Double-positive thymocytes	CD45^+^, TCRb^low^, CD4^+^, CD8^+^	↑↑	Dos Anj and os Cassado (2017)
8	Very small embryonic-like (VSEL) cells	CD117^-^, Ly6A/E^+^, CD4^−^, CD8^−^, CD11b^-^	↓	Kumar et al. (2008), Szade et al. (2013)
9	Double-positive thymocytes	CD45^+^, CD4^+^, CD8^+^	↑↑	Li et al. (2021)

### Effect of ascending infection-induced preterm birth on postpartum maternal cardiovascular function

**FIGURE 5:**
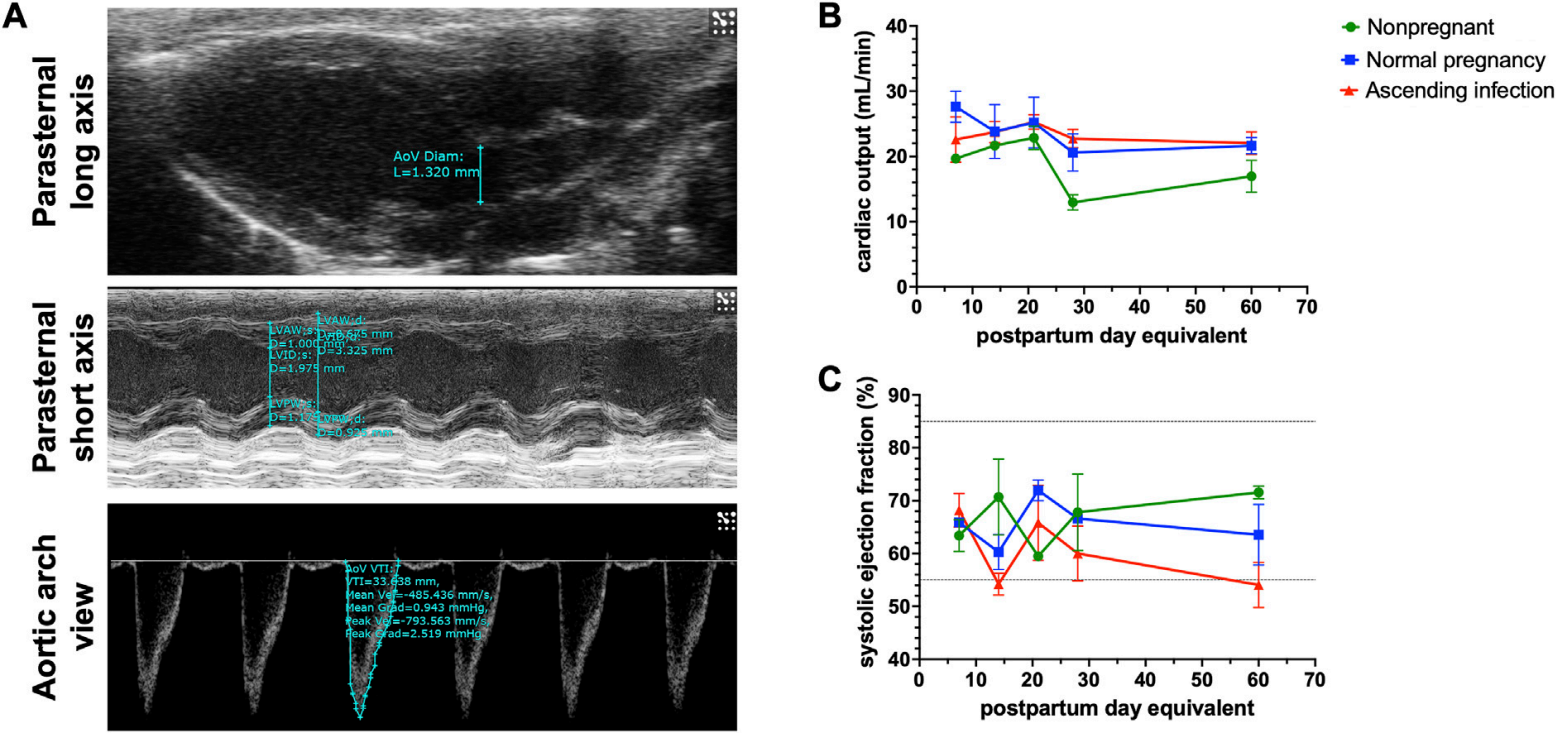
Postpartum cardiac physiologic parameters following normal pregnancy and ascending infection. **(A)** Echocardiographic measurements to determine cardiac output and systolic ejection fraction. **(B)** Postpartum cardiac output in normal pregnancy and ascending infection, with equivalent measurements for nonpregnant mice. **(C)** Postpartum systolic ejection fraction in normal pregnancy and ascending infection, with equivalent measurements for nonpregnant mice.

Supplementary Table S2 shows the baseline postpartum characteristics of the two pregnant mice groups. Of the 8 pregnant mice treated intravaginally with *E. coli*, 5 delivered live babies preterm, consistent with Spencer et al. (2021). Figure 5A shows various cardiac parameters measured via echocardiography up to 60 days postpartum. As shown in Figure 5B, there was no difference in overall postpartum cardiac output between normal pregnancy and ascending infection groups, with cardiac output decreasing to pre-pregnancy levels before postpartum day 60. Interestingly, when looking at ejection fraction which is indicative of systolic function or cardiac pumping (Figure 5C), the ascending infection group had lower ejection fraction at postpartum day 60, similar to that seen in peripartum cardiomyopathy (Bhattacharyya et al., 2012). However, the trend observed in these animals were not statistically significant, likely due to lack of power in our study.

## Discussion

Studying fetal microchimerism in humans and mice initially relied on the detection of SRY gene or Y chromosome present in male microchimeric cells (Bonney and Lantz, 1995; Bonney and Matzinger, 1997; Bonney and Onyekwuluje, 2003; Bonney and Onyekwuluje, 2004), which underestimates female microchimeric cells (Tan et al., 2005; Yan et al., 2006; Nemescu et al., 2016). With the development of Cre reporter mouse models, fetal microchimeric cells and extracellular vesicles can be easily detected via expression of a fluorescent protein, differentiating them from maternally-derived cells and tissues (Kara et al., 2012; Sheller-Miller et al., 2019). Our study was able to show the following: (1) fetal microchimeric cells can escape the maternal-fetal interface and migrate to other maternal organs, including maternal heart; (2) there were differences in fetal microchimeric cell populations, particularly fetal antigen-presenting cells (APCs), fetal hematopoietic stem cells and fetal double-positive thymocytes, in normal pregnancy and ascending infection; (3) fetal microchimeric immune cells can persist up to 28 days postpartum, with the persistent cell populations different between normal pregnancy and ascending infection; and (4) no significant changes in maternal cardiac physiology were observed between normal pregnancy and ascending infection groups 60 days postpartum. The trend to reduce ejection fraction that we observed in all animals with pathologic pregnancies is indicative of the development of peripartum cardiomyopathy (Bhattacharyya et al., 2012). We also speculate that if these animals are challenged long-term with a compound or risk factor of cardiac function, they are likely to develop cardiovascular disease usually seen in older cohort.

The primary purpose of the study is to characterize the cells in physiologic and pathologic pregnancies; hence, functional aspects were not pursued. One of the primary fetal microchimeric cell populations found in maternal hearts during pregnancy and postpartum was hematopoietic stem cells. The possible role of bone marrow-derived stem cells in cardiac regeneration has been a topic of investigation for many years (Chen et al., 2004; Wollert et al., 2004; Lunde et al., 2005; Janssens et al., 2006; Choudry et al., 2016). Earlier studies have reported regeneration of ischemic cardiac tissue by adult stem cells (Jackson et al., 2001; Orlic et al., 2001). These hematopoietic stem cells, however, do not readily transdifferentiate into myocytes (Murry et al., 2004). Interestingly, hematopoietic stem cells were found to generate cardiomyocytes at a low frequency via cell fusion (Alvarez-Dolado et al., 2003; Nygren et al., 2004), which could potentially contribute to cardiac reverse remodeling that happens postpartum. It is also possible that these fetally-derived progenitors can generate functional fetal microchimeric immune cells, such as seen in the maternal thymus of RAG^−/−^ female mice mated to congenic wild-type RAG^+/+^ mice (Khosrotehrani et al., 2008). In our study, we found two fetal microchimeric bone marrow-derived stem cell populations: LSK^+^ and LSK^−^ cells. While LSK^+^ cells comprise the multipotent hematopoietic cells found in the bone marrow (Challen et al., 2009), LSK^−^ cells do not have long-term repopulation capacity or myeloid potential (Kumar et al., 2008). Instead, these cells, also known as very small embryonice-like (VSEL) cells, are a heterogeneous cell population that contain early lymphoid-committed precursors distinct from common lymphoid progenitors (Kumar et al., 2008). VSEL cells do not express Oct4A and are found to have higher apoptotic rate compared to LSK^+^ cells, which could be important in the regulation of survival of hematopoietic and leukemic stem cells (Peng et al., 2012; Szade et al., 2013). In our high-dimensional single cell analysis, fetal microchimeric LSK^+^ and LSK^−^ hematopoietic stem cells did not persist in maternal hearts in ascending infection compared to normal pregnancy. Whether the contribution of decreased hematopoietic stem cells in maternal heart is vital enough to result in long-term cardiac changes that increase cardiovascular risk in preterm birth remains to be determined mechanistically.

Another fetal microchimeric cell population detected in our analysis was double-positive thymocytes. These immature thymocytes, which developed from double-negative thymocytes, express both CD4 and CD8 along with TCR, which comprise three-quarters of all thymocytes (Matsumoto et al., 1991; de Meis et al., 2012). These thymocytes then undergo positive selection wherein they escape apoptosis to become either CD4^+^ or CD8^+^ single-positive T cells (Savino et al., 2007). In response to bacterial or viral infection or stress, double-positive cells usually undergo apoptosis resulting in decreased thymic cellularity and atrophy (Dooley and Liston, 2012). However, during pregnancy, escape of double-positive fetal thymocytes into the maternal periphery was observed instead, eventually settling down in maternal heart. This phenomenon of thymocyte escape was observed more in parasitic infections such as *Trypanosoma cruzi* (Mendes-da-Cruz et al., 2003; Mendes-da-Cruz et al., 2006), *Plasmodium berghei* (Francelin et al., 2011) or *Schistosoma mansoni* (Wellhausen and Boros, 1982) than in bacterial or viral infections. Interestingly, decreased frequency of double-positive fetal thymocytes in maternal heart was initially observed with ascending infection. These fetal thymocytes, however, were cleared more following normal pregnancy, resulting in their persistence following an ascending infection. How double-positive thymocytes affect cardiac microenvironment remains to be unknown, although a subset of these double-positive cells was associated with severe cardiac forms of Chagas disease, pointing to a possible autoimmune pathology (Morrot et al., 2011).

Other fetal microchimeric immune cells detected in maternal heart were macrophages and dendritic cells. Both cell types form the mononuclear phagocyte system that is important in maintaining homeostasis in the cardiac tissue (Van der Borght and Lambrecht, 2018).These cells respond in response to ischemic injury and inflammation, ultimately resulting in fibrosis that can promote cardiac remodeling (Simões and Riley, 2022). To limit formation of fibrotic scar, there must be less conventional dendritic cells and macrophages during the cardiac repair process. In particular, we detected fetally CD11c+ macrophages in maternal heart, which has been implicated in the pathogenesis of acute coronary arteritis (Motomura et al., 2015). In our postpartum data, higher fetal conventional dendritic cells and CD11c+ macrophages were seen in ascending infection, which may lead to cardiac remodeling especially in the presence of a stressor that can cause cardiac injury. These antigen-presenting cells, along with CD25^+^ B cells which were thought to be memory B cells (Amu et al., 2010), may present to maternal T cells via direct allorecognition (i.e., direct MHC presentation by fetal APCs) or semi-direct alloregonition (e.g., recycling and expression of fetal MHC by maternal APCs) (Murrieta-Coxca et al., 2022). Whether this presentation is tolerogenic or stimulatory up to the point of autoimmune reaction needs further scrutiny of co-stimulatory interactions between these APCs and maternal T cells.

With our reporter model, we were able to detect fetal cells in not just in maternal heart but also in various maternal organs such as lungs and kidneys (Supplementary Figure S1), consistent with previous reports (O'Donoghue, 2008). While this suggests dissemination of these cells *via* maternal circulation, it does not necessarily imply that the responses seen were due to a more global phenomenon such as systemic inflammation. There is increasing evidence that these cells selectively, not randomly, home to various maternal tissues where they affect the local tissue microenvironment (Kara et al., 2012; Seppanen et al., 2012). The differences seen in immune cell subpopulations in maternal hearts depending on the context of pregnancy (physiologic vs. pathologic) add further evidence to this selective trafficking of fetal microchimeric cells. How these fetal microchimeric cells interact with the maternal immune system, especially in lymphoid tissues such as bone marrow, spleen and thymus, to modulate this selective fetal cell trafficking requires further scrutiny.

Whether these microchimeric cells would persist or be cleared by the maternal immune system would depend on either dysregulation of apoptosis in fetal cells (Kolialexi et al., 2004) or the interaction between fetal cells and the maternal immune cells in various immune interfaces (Gammill and Nelson, 2010). Initially these cells are rapidly cleared within 1 month of delivery in mice (Bonney and Matzinger, 1997), followed by a decrease in rate of clearance resulting in long-term persistence (Pritchard et al., 2012). Those that persist could help with cardiac repair (i.e., beneficial) or result in autoimmune reaction (i.e., detrimental), which would depend on the context of pregnancy and what cell types persist after. Interestingly, in a prospective cohort study looking into the impact of male-origin fetal microchimerism on cardiovascular risk, microchimerism was associated with decreased risk of ischemic heart disease (Hallum et al., 2021), which supports that pregnancy can be beneficial to maternal cardiac health, provided that the pregnancy was not negatively impacted by disease etiologies such as preterm birth or preeclampsia.

Given that the fetus is considered a semi-allogeneic “graft” during pregnancy, employing syngeneic mating (i.e., mating female wild-type C57BL/6J with male homozygous mT + C57BL/6J) poses a major limitation to this study. Syngeneic mating results in major histocompatibility complexes and other proteins and peptides being shared between the mother and the progeny (considered as “non-foreign”) (Prell et al., 2016), thus lacking the maternal allo-antibodies to most paternally-derived fetal antigens usually seen in pregnancy. However, syngeneic mating does not rule out the possibility of a maternal immune response to fetal antigens as H-Y antigens, which are a group of Y chromosome-encoded minor histocompatibility antigens (Popli et al., 2014), can induce response from the maternal immune system during pregnancy (Bonney and Onyekwuluje, 2003; Bonney and Onyekwuluje, 2004). Another major limitation of our study was it only investigated changes in frequency of various fetal microchimeric immune cell subpopulations in maternal hearts. The number of microchimeric cells found in maternal hearts is known to increase with insults such as myocardial infarction (Kara et al., 2012), hence looking at both changes in overall cell numbers and composition, and how they affect the overall cardiac microenvironment would provide stronger evidence of the impact of fetal microchimerism on maternal health. Our study was also not able to fully characterize fetal microchimeric cells of non-hematopoietic origin due to limitations in the antibody panel used, which mainly allowed characterization of cells of hematopoietic origin. This population is equally important because they may contain stem cells that ultimately contribute to cardiac repair and remodeling. Using a similar reporter system, Kara et al. (2012) were able to show that in a surgical model of maternal myocardial infarction, fetal cells can selectively home to sites of cardiac injury where they undergo differentiation into diverse cardiac cell types. These fetal cells express *Cdx2*, which was previously associated with cells derived from trophectoderm. Further studies have also shown that mesenchymal stem cells of non-hematopoietic origin can instead be mobilized to differentiate cardiomyocytes (Kawada et al., 2004; Fukuda and Fujita, 2005), thus highlighting the potential significance of characterizing the CD45^−^ fetal microchimeric cells. This antibody panel does not also characterize different B cell subpopulations, as certain subsets such as B10 cells have been implicated in less fibrosis during cardiac repair (Wu et al., 2019; Chen et al., 2022). Aside from the limitations intrinsic to the antibody panel used, another limitation was the sample size and duration of observation for the echocardiographic studies. Given that only 9 animals (3 per treatment group) were used, statistical power was not sufficient to establish statistical significance. The animals were also monitored for only 60 days postpartum, which may be sufficient to observe short- and medium-term cardiac changes, but not long-term changes. It could also be possible that differences in cardiac physiology would be more apparent in the presence of a stressor (e.g., isoproterenol-induced myocardial injury) (Brooks and Conrad, 2009; Krenek et al., 2009).

In conclusion, different compositions of fetal microchimeric immune cells can migrate and persist in maternal heart, depending on the context of pregnancy (i.e., term or ascending infection-associated preterm delivery). These cells have the potential to modulate the maternal cardiac tissue microenvironment, but whether they can only affect short-term outcomes (e.g., reverse cardiac remodeling during postpartum) or long-term outcomes (e.g., future maternal cardiovascular risk) needs further mechanistic studies.

## Data Availability

The original contributions presented in the study are included in the article/Supplementary Material, further inquiries can be directed to the corresponding authors.
