# Supplementary figures and images for: Characterization of fetal microchimeric immune cells in mouse maternal hearts during physiologic and pathologic pregnancies
Source: Front Cell Dev Biol. 2023 Sep 22;11:1256945. doi: 10.3389/fcell.2023.1256945 (PMC10556483; doi:10.3389/fcell.2023.1256945)

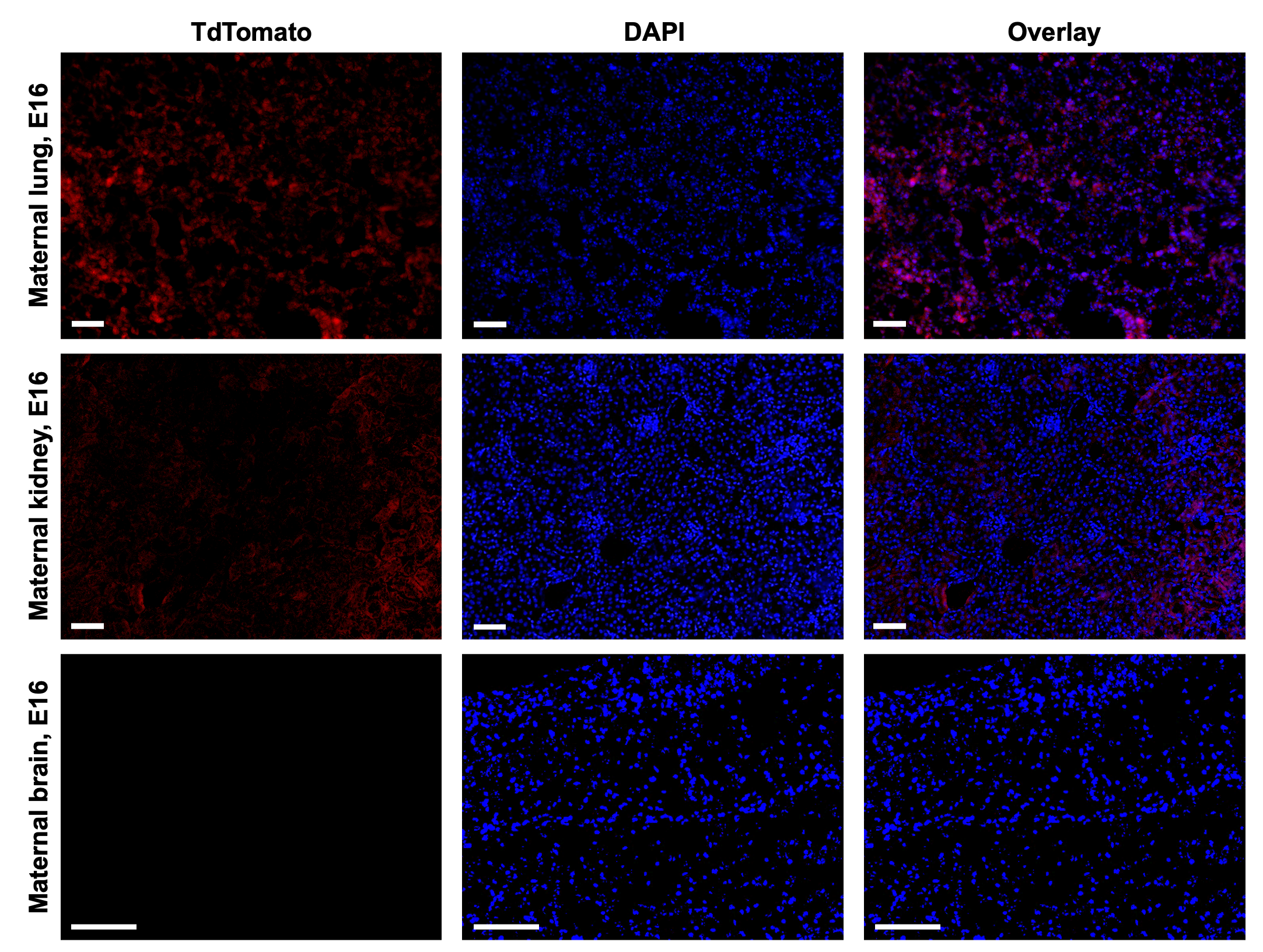

Supplement: Supplementary file 1 [file Image1.tiff]
