# Supplementary material for: Characterization of fetal microchimeric immune cells in mouse maternal hearts during physiologic and pathologic pregnancies
Source: Front Cell Dev Biol. 2023 Sep 22;11:1256945. doi: 10.3389/fcell.2023.1256945 (PMC10556483; doi:10.3389/fcell.2023.1256945)
Supplement: Supplementary file 2 [file Table1.docx]

**Supplementary Table 1. List of antibodies used for mass cytometry.**

| **Tagged Ab Description** | **Target** | **Label** | **Clone** | **Specificities** | **Source** | **Catalog No.** |
| --- | --- | --- | --- | --- | --- | --- |
| CD45 89Y | CD45 | ^89^Y | 30-F11 | Ms | DVS-Fluidigm | 3089005B |
| CD4 115In | CD4 | ^115^In | RM4-5 | Ms | BioLegend | 100506 |
| CD11c 142Nd | CD11c | ^142^Nd | N418 | Ms | DVS-Fluidigm | 3142003B |
| TCRb 143Nd | TCRb | ^143^Nd | H57-597 | Ms | DVS-Fluidigm | 3143010B |
| GATA3 145Nd | GATA3 | ^145^Nd | TWAJ | Hu, Ms, Pg, Rh | eBioscience | 14-9966-82 |
| CD8a 146Nd (MDA) | CD8a | ^146^Nd | 53-6.7 | Ms | BioLegend | 100702 |
| CD11b 148Nd | CD11b | ^148^Nd | M1/70 | Ms, Hu | DVS-Fluidigm | 3148003B |
| CD19 149Sm (MDA) | CD19 | ^149^Sm | 4D5 | Ms | BioLegend | 115502 |
| CD25 150Nd (MDA) | CD25 | ^150^Nd | 3C7 | Ms | BioLegend | 101902 |
| CD86 152Sm | CD86 | ^152^Sm |  | Ms | BioLegend | In-house conjugation |
| T-bet 154Sm | T-bet | ^154^Sm | 4B10 | Hu, Ms | BioLegend | 644825 |
| CD14 156Gd | CD14 | ^156^Gd | Sa14-2 | Ms | DVS-Fluidigm | 3156009B |
| Foxp3 158Gd | Foxp3 | ^158^Gd | FJK-16s | Ms, Rt, Bv, Cn, Po, Fe | DVS-Fluidigm | 3158003A |
| F4/80 159Tb (MDA) | F4/80 | ^159^Tb | BM8 | Ms | BioLegend | 123102 |
| PU.1 161Dy | PU.1 | ^161^Dy | 7C2C34 | Ms | BioLegend | 681302 |
| Ly-6A/E 164Dy | Ly-6A/E, Sca-1 | ^164^Dy | D7 | Ms | DVS-Fluidigm | 3164005B |
| IFNg 165Ho | IFNg | ^165^Ho | XMG1.2 | Ms | DVS-Fluidigm | 3165003B |
| IL-4 166Er | IL-4 | ^166^Er | 11B11 | Ms | DVS-Fluidigm | 3166003B |
| CD117 169Tm | CD117 | ^169^Tm | 2B8 | Ms | BioLegend | 105802 |
| NK1.1 170Er | NK1.1, CD161b/c, Ly-55 | ^170^Er | PK136 | Ms | DVS-Fluidigm | 3170002B |
| Siglec-F 172Yb | Siglec-F | ^172^Yb | E50-2440 | Ms | BD | 552125 |
| IL-17A 174Yb | IL-17A | ^174^Yb | TC11-18H10.1 | Ms | DVS-Fluidigm | 3174002B |
| TdTomato 175Lu | TdTomato | ^175^Lu |  | Ms | BioLegend | In-house conjugation |
| I-A/I-E 209Bi | I-A/I-E, MHC-II | ^209^Bi | M5/114.15.2 | Ms | DVS-Fluidigm | 3209006B |
