# Supplementary material for: Characterization of fetal microchimeric immune cells in mouse maternal hearts during physiologic and pathologic pregnancies
Source: Front Cell Dev Biol. 2023 Sep 22;11:1256945. doi: 10.3389/fcell.2023.1256945 (PMC10556483; doi:10.3389/fcell.2023.1256945)
Supplement: Supplementary file 3 [file Table2.docx]

**Supplementary Table 2. Baseline postpartum characteristics of pregnant mice groups.**

|  | Ascending infection (n=8) | Normal pregnancy (n=8) | p-value |
| --- | --- | --- | --- |
| Mean gestational period (d) | 17.75 ± 1.16 | 19.13 ± 0.64 | 0.011 |
| Average litter size | 5.62 ± 0.74 | 5.50 ± 0.76 | 0.744 |
| Number of pups alive after weaning / birthing mother | 5.62 ± 0.74 | 5.50 ± 0.76 | 0.744 |
